# Differential Impact of Adolescent or Adult Stress on Behavior and Cortical Parvalbumin Interneurons and Perineuronal Nets in Male and Female Mice

**DOI:** 10.1093/ijnp/pyae042

**Published:** 2024-09-14

**Authors:** Thamyris Santos-Silva, Beatriz Kinchin Souza, Débora Akemi Endo Colodete, Lara Ramos Campos, Thaís Santos Almeida Lima, Francisco S Guimarães, Felipe V Gomes

**Affiliations:** Department of Pharmacology, Ribeirão Preto Medical School, University of São Paulo, Ribeirão Preto, Brazil; Department of Pharmacology, Ribeirão Preto Medical School, University of São Paulo, Ribeirão Preto, Brazil; Department of Pharmacology, Ribeirão Preto Medical School, University of São Paulo, Ribeirão Preto, Brazil; Department of Pharmacology, Ribeirão Preto Medical School, University of São Paulo, Ribeirão Preto, Brazil; Department of Pharmacology, Ribeirão Preto Medical School, University of São Paulo, Ribeirão Preto, Brazil; Department of Pharmacology, Ribeirão Preto Medical School, University of São Paulo, Ribeirão Preto, Brazil; Department of Pharmacology, Ribeirão Preto Medical School, University of São Paulo, Ribeirão Preto, Brazil

**Keywords:** GABA interneurons, sex differences, individual variability, neurodevelopment

## Abstract

**Background:**

Stress has become a common public health concern, contributing to the rising prevalence of psychiatric disorders. Understanding the impact of stress considering critical variables, such as age, sex, and individual differences, is of the utmost importance for developing effective intervention strategies.

**Methods:**

Stress effects (daily footshocks for 10 days) during adolescence (postnatal day [PND] 31–40) and adulthood (PND 65–74) were investigated on behavioral outcomes and parvalbumin (PV)-expressing GABAergic interneurons and their associated perineuronal nets (PNNs) in the prefrontal cortex of male and female mice 5 weeks post stress.

**Results:**

In adulthood, adolescent stress induced behavioral alterations in male mice, including anxiety-like behaviors, social deficits, cognitive impairments, and altered dopamine system responsivity. Applying integrated behavioral z-score analysis, we identified sex-specific differences in response to adolescent stress, with males displaying greater vulnerability than females. Furthermore, adolescent-stressed male mice showed decreased PV+ and PNN+ cell numbers and PV+/PNN+ colocalization, while in females, adolescent stress reduced prefrontal PV+/PNN+ colocalization in the prefrontal cortex. Further analysis identified distinct behavioral clusters, with certain females demonstrating resilience to adolescent stress-induced deficits in sociability and PV+ cell number. Adult stress in male and female mice did not cause long-lasting changes in behavior and PV+ and PNN+ cell number.

**Conclusion:**

Our findings indicate that the timing of stress, sex, and individual variabilities seem to be determinants for the development of behavioral changes associated with psychiatric disorders, particularly in male mice during adolescence.

**Significance Statement**

Comprehending the effects of stress exposure at distinct developmental stages on males and females is crucial in tackling the increasing incidence of psychiatric diseases. This study shows that adolescent—but not adult—stress leads to long-lasting behavioral and neurobiological alterations in male mice, including increased anxiety, social deficits, and disruptions in prefrontal parvalbumin interneurons and their surrounding perineuronal nets. In contrast, the adolescent stress response in female mice varied, with some showing resilience to develop social interaction and prefrontal parvalbumin interneurons. By revealing sex-specific variations in stress impact on behavioral outcomes neuroplastic markers, our results emphasize the need to consider age- and sex-dependent factors when investigating stress-related disorders. This study also provides critical insights into understanding different trajectories that males and females may show in stress-related diseases and offers a foundation for creating more efficient and customized therapeutic strategies.

## INTRODUCTION

In our rapidly changing world, individuals are facing heightened levels of stress (Almeida et al., 2020). Alongside, psychiatric disorder prevalence is rising, including stress-related mood disorders, impacting individuals across all age groups and genders (Whiteford et al., 2015; Santomauro et al., 2021). The impact of stressors greatly depends on the developmental stage (Gomes et al., 2020). During adolescence, a period of high neuroplasticity, brain structures are remodeled and neuronal circuits are maturated, which makes them particularly sensitive to socioenvironmental stressors (Hensch, 2005; Gomes et al., 2016, 2019). While adulthood lacks the same level of plasticity, the brain remains vulnerable to stress due to neurocircuit changes that persist throughout life (McEwen and Morrison, 2013). Sex-specific differences in psychiatric disorder prevalence and outcomes highlight that stress response varies in male and female individuals (Bangasser et al., 2018). Furthermore, stress and behavioral responses differ among individuals due to genetic, environmental, and psychosocial factors, facilitating coping and resilience or increasing susceptibility to the emergence of psychiatric disorders (Ebner and Singewald, 2017; Santos-Silva et al., 2023). Therefore, understanding age and sex differences, as well as intrinsic interindividual variability, is imperative for effectively addressing mental health challenges associated with stress.

A particular brain region sensitive to stress is the prefrontal cortex (PFC) (McEwen and Morrison, 2013), which only completely develops in late adolescence or early adulthood (Chini and Hanganu-Opatz, 2021). Stress exposure disrupts higher cognitive functions and social processes, which are critical aspects of PFC function (Arnsten, 2015; Friedman and Robbins, 2021) and are impaired in several psychiatric disorders (Gomes and Grace, 2017; Dorofeikova et al., 2023). The PFC shows a rich population of fast-spiking GABAergic interneurons containing the calcium-binding protein parvalbumin (PVIs). These interneurons are critical in regulating the excitatory-inhibitory balance within cortical circuits (Hu et al., 2014).

PVIs are highly sensitive to stress, and their dysfunction has been implicated in several psychiatric disorders, including stress-related mood disorders and schizophrenia (Perlman et al., 2021; Santos-Silva et al., 2024b), which are known to exhibit age- and sex-dependent differences in prevalence and symptomatology (Otte et al., 2016; Sommer et al., 2020; Solmi et al., 2021). Around 60% of PVIs are wrapped by the perineuronal nets (PNNs) (Lupori et al., 2023), a specialized extracellular matrix that protects them from oxidative stress (Cabungcal et al., 2013) and modulates their function and maturation by several mechanisms (Santos-Silva et al., 2024a). Age and sex differences have been observed in the stress responsiveness of PVIs and PNNs, along with behavioral changes (Woodward and Coutellier, 2021). For example, we previously showed that rats, in a period corresponding to adolescence, exhibited heightened vulnerability to stress-induced modifications compared with their adult counterparts, potentially due to PVI and PNN maturation during sensitive periods of plasticity (Gomes et al., 2020; Colodete et al., 2024). Moreover, stress-induced impairments in learning are regulated by PVIs in a sex-dependent manner (Binette et al., 2023). These findings suggest that the interaction between stress, age, and sex influences the integrity of PNNs and PVIs, potentially contributing to the development of psychiatric disorders with distinct age and sex patterns.

Here, we explored behavioral outcomes and neurobiology mechanisms mediating the effect of stress in the PFC, considering age and sex. We first investigated the impact of stress during adolescence and adulthood in male and female mice within distinct domains: anxiety, sociability, cognitive function, and dopamine system responsivity. Then, we evaluated stress-induced changes in cortical PVIs and PNNs and examined their relationship with behavioral outcomes. Lastly, we investigated intrinsic interindividual variability in stress response to identify resilient and susceptible animals. Altogether, our findings underscore the importance of considering age and sex differences in stress response, with potential implications for understanding divergent stress outcomes among males and females.

## MATERIALS AND METHODS

A detailed description of all experimental procedures is provided in the Supplementary Material.

### Animals

Male and female C57BL/6 mice (postnatal day [PND]24 or 58) were housed (2–4 animals per cage) in a temperature- (22°C) and humidity- (47%) controlled environment (12-hour-light/-dark cycle) with water and food ad libitum. All procedures were approved by the Ribeirão Preto Medical School Ethics Committee (#100/2019).

### Stress Protocol

Mice were exposed to daily inescapable footshock (FS) for 10 days during adolescence (PND31–40) or adulthood (PND65–74). Naïve animals were left undisturbed in their home cages.

### Behavioral Characterization

Five weeks after the stress protocol, mice were tested in the following behavioral tests: elevated plus maze (EPM; on PND75 [post-adolescent stress] or PND110 [post-adult stress]); social interaction (SI) and social discrimination test (on PND76 [post-adolescent stress] or PND111 [post-adult stress]); novel-object recognition test (NOR; on PND77 [post-adolescent stress] or PND112 [post-adult stress]); and amphetamine-induced hyperlocomotion (AIH; on PND78 [post-adolescent stress] or PND113 [post-adult stress]). A detailed description of EPM, SI, social discrimination, NOR, and AIH is provided in the Supplementary Material.

#### Behavioral z-Score Index

***—*** The raw data for the EPM parameters (%time spent and entries into the open-arms and number of closed-arms entries), SI index, social discrimination index, NOR index, and AIH was normalized using z-normalized data, as the following equation,


$$\begin{array}{l} z={\;}^{\left(x-mean\;of\;na\ddot{i}ve\;group\right)}{/}_{standard\;deviation\;of\;na\ddot{i}ve\;group} \end{array}$$

in which x represents the individual raw data for the observed parameter.

Then, the integrated behavioral z-score was obtained by averaging the EPM, SI, social discrimination, NOR, and AIH z-normalized data, as previously described (Guilloux et al., 2011).

### Immunofluorescence

All immunofluorescence procedures, image acquisition, and analysis followed previously reported protocol with minor modifications (Gomes et al., 2020; Santos-Silva et al., 2024c) and are provided in the Supplementary Material.

### Statistical Analyses

Data were presented as mean ± SEM and analyzed using Student test and 1-, 2-, or 3-way ANOVA accordingly. Significant differences were indicated by *P *< .05.

## RESULTS

### Stress Affects Sociability, Memory Cognition, and Dopamine System Responsivity in an Age- and Sex-Dependent Manner

**Figure 1.**
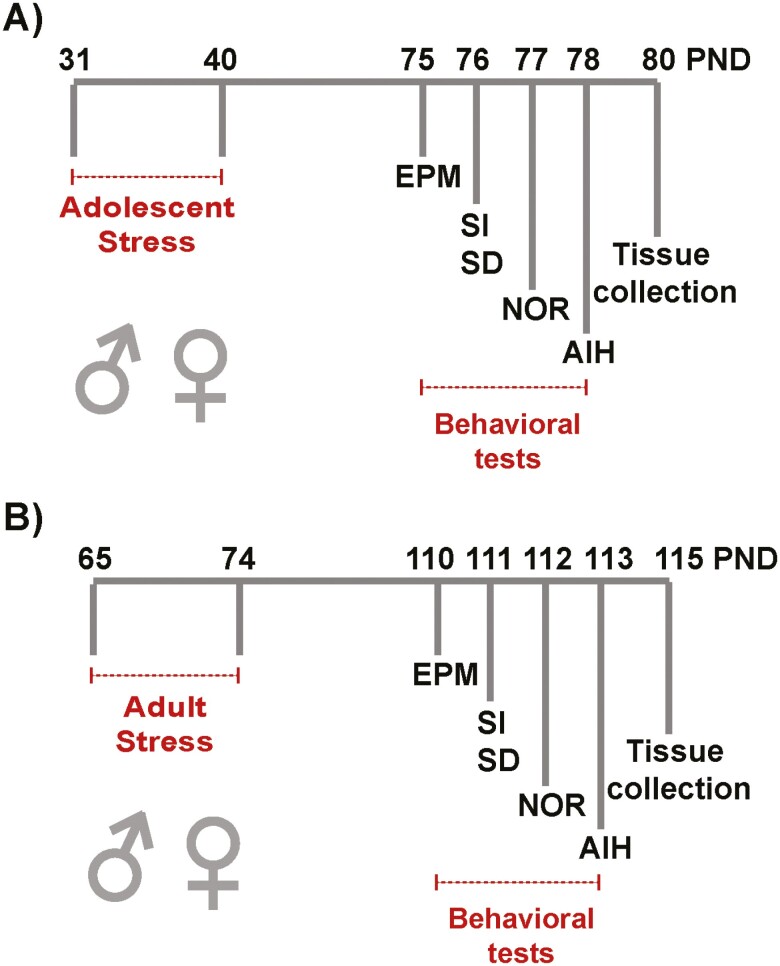
Experimental timeline. (A) Adolescent mice (male and female) were stressed via inescapable footshocks from postnatal day (PND)31 to 40. At adulthood, from PND 75 to 78, animals were subjected to elevated plus maze (EPM), social interaction (SI), social discrimination (SD), novel object recognition (NOR), and amphetamine-induced hyperlocomotion (AIH) tests (n = 7–10 naïve and 7–10 stressed). Prefrontal cortex (PFC) tissue was collected on PND80. (B) Adult mice (male and female) were stressed via inescapable footshocks from PND65 to 74. From PND110 to 113, animals were tested in EPM, SI, social discrimination, NOR, and AIH (n = 9–10 naïve and 9–10 stressed). PFC tissue was collected on PND115.

We aimed to characterize the impact of stress during adolescence (PND31–40) or adulthood (PND65–74) in male and female mice. At adulthood, adolescent- and adult-stressed mice and naïve groups were subject to behavioral tests to evaluate anxiety, sociability, cognitive function, and dopamine system response 5 weeks post-stress (post-adolescent stress: PND75–78; post-adult stress: PND110–113), followed by PFC collection (post-adolescent stress: PND80, or post-adult stress: PND115; Figure 1A, B). First, we evaluated the effects of sex and interactions between sex and stress and the timing of stress for all behavioral parameters using 3-way ANOVA. There was a significant sex effect in basal locomotor activity and AIH but no interactions among sex, stress, and timing of stress (supplementary Table 1).

**Figure 2.**
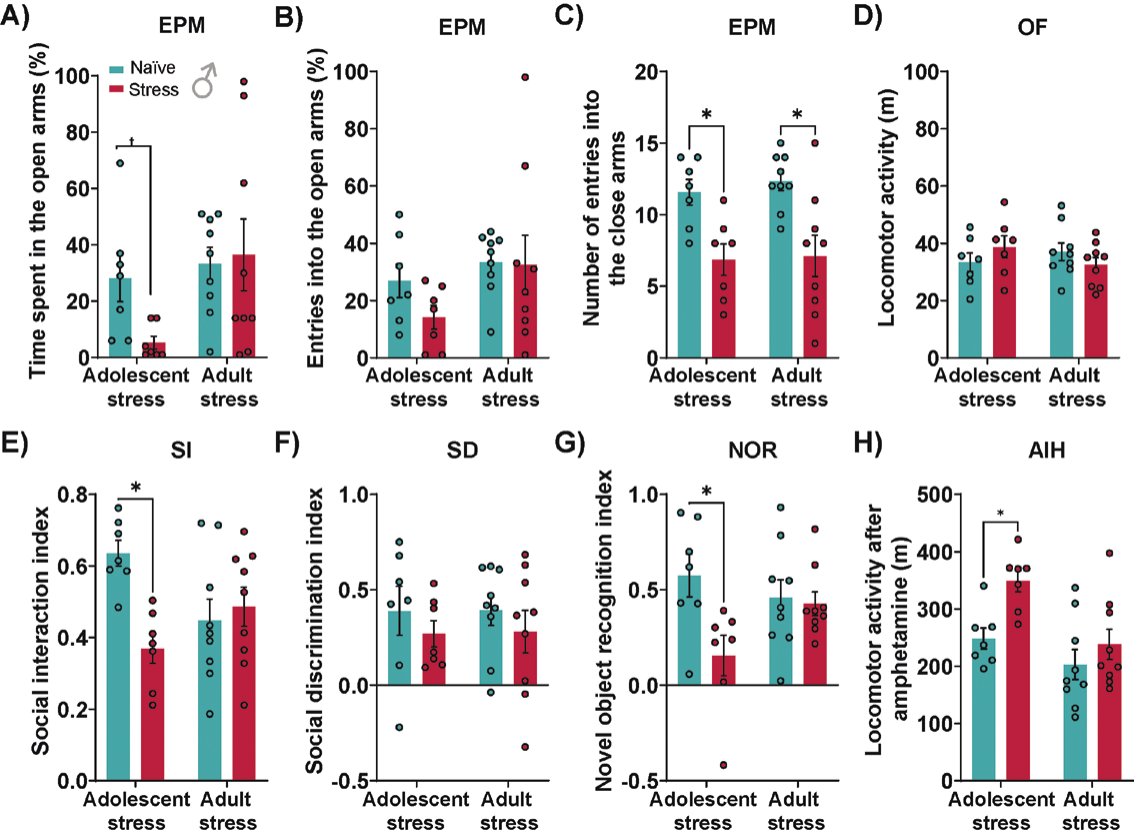
Effects of adolescent and adult stress on male mice behavior. Stress during adolescence reduced (A) percent of time spent in the open arms but not (B) percent of entries in the open arms of the elevated plus maze (EPM). (C) Adolescent and adult stress decreased the number of entries into the closed arms of the EPM. (D) In the open field (OF), no change was observed in total locomotor activity. (E) Adolescent stress negatively impacted the social interaction (SI) index (F) without affecting the social discrimination (SD) index. Additionally, (G) adolescent stress, but not adult stress, decreases the novel object recognition (NOR) index. (H) Amphetamine-induced hyperlocomotion (AIH) increased in male mice exposed to adolescent stress. n = 7–9/group. Data are shown as mean ± SEM. t = *P* < .05 from unpaired Student *t* test; *P < .05, 2-way ANOVA followed by Tukey post-test.

In male mice, 2-way ANOVA indicated an effect of timing of stress in the percent of time spent in the open arms (F_1,28 _= 4.21, *P *= .04), without an effect of stress or interaction between factors. Post-hoc Tukey test revealed no changes in the percent of time spent in the open arms of stressed animals compared with their naïve counterparts (Figure 2A). However, by performing an unpaired Student *t* test, the percent of time spent in the open arm was reduced in males exposed to adolescent stress (t_12 _= 2.66; *P *= .02), indicating an anxiety-like behavior. No change was found in the percent of open-arms entries (Figure 2B). Similar to our previous studies in rats (Cavichioli et al., 2023; Santos-Silva et al., 2024c), adolescent and adult stress decreased the number of closed-arms entries (*P *< .05 vs naïve; Tukey post-test) (Figure 2C). A 2-way ANOVA indicated a stress effect (F_1,28 _= 20.50, *P *= .0001) but no effect of timing of stress and interaction between these factors. To ensure that the reduced number of closed-arms entries would reflect an anxiety-like behavior instead of impairments in locomotion, we evaluated the locomotor activity in the open field (OF). Males exposed to adolescent or adult stress did not show changes in the distance traveled in the OF (Figure 2D). Confirming the anxiety-like behavior observed in the EPM, the locomotor distance in the center zone of OF was reduced in adolescent-stressed male mice (supplementary Figure 1A). Regarding sociability, a 2-way ANOVA showed an effect of stress (F_1,28_ = 4.80, *P* = .04) and an interaction between stress and timing of stress for the SI index (F_1,28_ = 8.54, *P* = .006), but with no effect of timing of stress. Post-hoc analysis revealed a decrease in the SI index in mice subject to adolescent stress (*P* < .05 vs naïve; Tukey post-test; Figure 2E). No changes in the social discrimination index were caused by the adolescent and adult stress in male mice (Figure 2F). In the NOR, a 2-way ANOVA showed an effect of stress (F_1,28_ = 5.87, *P* = .02) and interaction between stress and timing of stress (F_1,28_ = 4.38, *P* = .04), with no effect of timing of stress exposure. Post-hoc analysis revealed that adolescent stress impaired object recognition (*P* < .05 vs naïve; Tukey post-test). In the AIH test, a 2-way ANOVA indicated an effect of stress (F_1,28_ = 8.00, *P* = .008) and timing of stress (F_1,28_ = 10.51, *P* = .003) without interaction between them. Post-hoc analysis indicated an increase in the locomotor activity induced by amphetamine in adolescent-stressed male mice (*P* < .05 vs naïve, Tukey post-test; Figure 2H), indicating an enhanced dopamine system responsivity caused by the adolescent stress.

**Figure 3.**
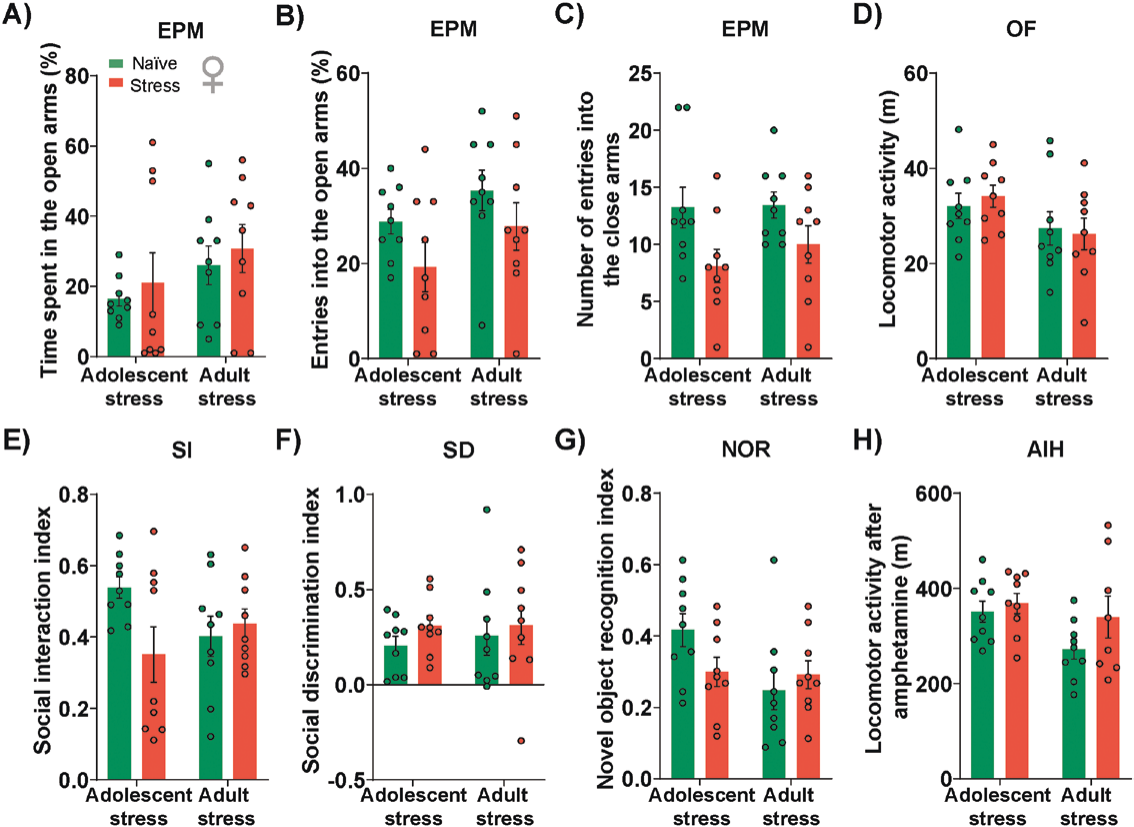
Effects of adolescent and adult stress on female mice behavior. Stress during adolescence or adulthood did not change (A) the time spent in the open arms, (B) entries into the open arms, and (C) the number of entries into the closed arms of the elevated plus maze (EPM). (D) No change in the distance traveled in the open field (OF) was found in the stressed animals. Additionally, no impact was observed in female mice subjected to (E) social interaction (SI) and (F) social discrimination (SD) tests 5 weeks after adolescent or adult stress. (G) No stress effect was observed in the novel object recognition (NOR) index of animals exposed during adolescence or adulthood. (H) Amphetamine-induced hyperlocomotion (AIH) was not changed in female mice exposed to adolescent or adult stress; n = 9/group. Data are shown as mean ± SEM. Two-way ANOVA followed by Tukey post-test.

Unlike the impact of stress in male mice, females exposed to adolescent or adult stress did not show anxiety-like behavior as observed in the percent of time spent in the open arms (Figure 3A), percent of open-arms entries (Figure 3B), and the number of closed-arms entries in the EPM (Figure 3C). In the OF, no alterations in the locomotor activity (Figure 3D) and exploration of the center zone (supplementary Figure 1B) were observed in females exposed to adolescent or adult stress. Additionally, adolescent and adult stress did not impact sociability (Figure 3E–F), cognitive function (Figure 3G), or AIH (Figure 3H). These findings indicate that female mice seem less susceptible to the long-lasting changes caused by adolescent and adult stress.

### Behavioral z-Score Revealed a Distinct Adolescent Stress Impact on Male and Female Mice

**Figure 4.**
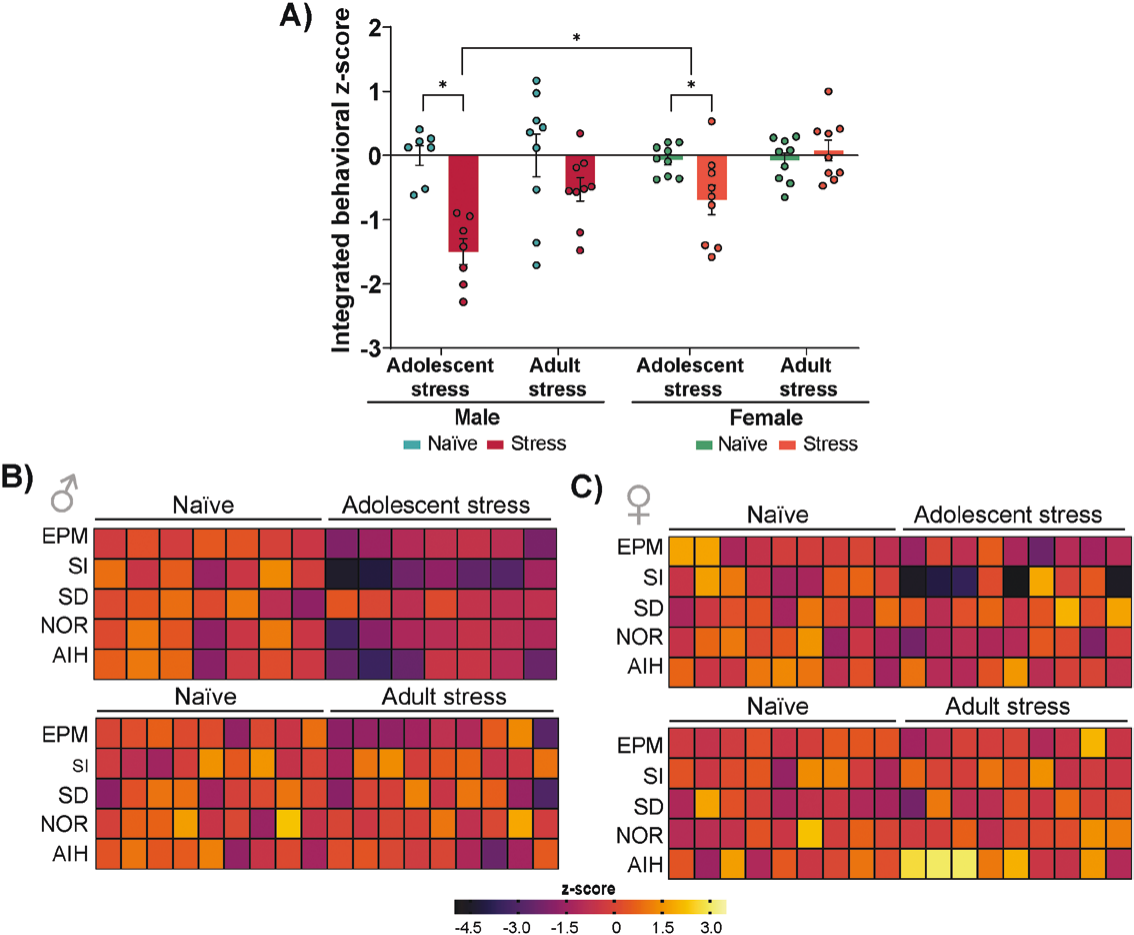
Behavioral z-score highlighted differences in the effects of adolescent stress on male and female mice. (A) Integrated behavioral z-score. (B) The heatmap of each behavioral z-score indicates the impact of stress during adolescence or adulthood on male and (C) female mice; n = 7–9/group. Data are shown as mean ± SEM. *P < .05; 3-way ANOVA followed by Tukey post-test.

To compare behavior changes across sexes (male vs female), we applied the integrated behavioral z-score, a statistical method that standardized the data (Guilloux et al., 2011). By combining the z-normalized behavioral parameters from all tests into a single index (integrated behavioral z-score), a 3-way ANOVA revealed a significant effect of stress (F_1,60_ = 5.06, *P* = .03), timing of stress (F_1,60_ = 9.34, *P* = .003), and sex (F_1,60_ = 19.46, *P* < .0001), as well as an interaction between stress and sex (F_1,60_ = 7.52, *P* = .008) and timing of stress and sex (F_1,60_ = 9.51, *P* = .003; Figure 4A). Post-hoc analysis revealed that adolescent stress impaired the integrated behavioral z-score in both males and females (*P* < .05 vs naïve of their respective groups; Tukey post-test). Male mice exposed to adolescent stress were more affected than females (*P* < .05; adolescent-stressed male mice vs adolescent-stressed female mice; Tukey post-test). A heatmap displaying z-normalized behavioral data of male mice confirmed a homogenous detrimental impact of adolescent stress in all behaviors (Figure 4B). The z-normalized behavioral data of female mice exposed to adolescent stress suggests an intra-group variability, especially in the SI index (Figure 4C). Conversely, adult stress in both male and female mice displayed similar heatmap of each behavioral z-score compared with their respective naïve groups (Figure 4B–C).

### Adolescent Stress Impacts Cortical PVIs and Their Associated PNNs

PVIs are more vulnerable to stress-induced damage in periods when PNNs are immature (Gomes et al., 2019a). Therefore, we evaluated the impact of stress on PV+ and PNN+ cell numbers and PV+/PNN+ colocalization in the prelimbic subregion of the PFC during adolescence, a sensitivity period of plasticity (when PVIs and PNNs are not completely developed), and adulthood (when PVIs and PNNs are completely developed) (Reichelt et al., 2019). A 3-way ANOVA showed a significant effect of sex on PV+ and PNN+ cell numbers and PV+/PNN+ colocalization, as well as interactions between sex and timing of stress in the number of PNN+ cells and PV+/PNN+ colocalization and among sex, stress, and timing of stress in the number of PNN+ cells (supplementary Table 2).

**Figure 5.**
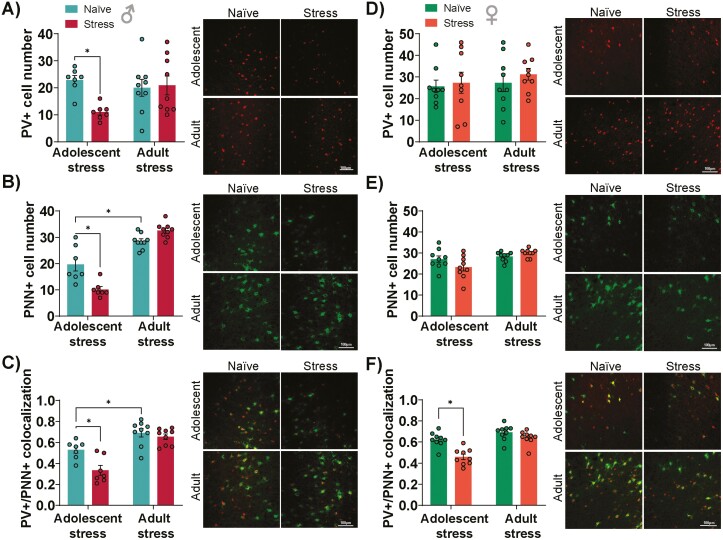
Impact of adolescent and adult stress on the number of parvalbumin (PV)+ and perineuronal nets (PNN)+ cells, and PV+/PNN+ colocalization in the prefrontal cortex (PFC). In male mice, adolescent stress (A) reduced the number of PV+, (B) PNN+, and (C) PV+/PNN+ colocalization in the PFC. Adolescent stress in female mice did not change (D) PV and (E) PNN markers. (F) Female mice stressed during adolescence showed decreased PV+/PNN+ colocalization in the PFC, as indicated by the Menders overlap coefficient. Representative images illustrating PV and PNN expression and their colocalization are placed on the left side of each graph; n = 7–9/group. Data are shown as mean ± SEM. *P < .05; 2-way ANOVA followed by Tukey post-test.

In male mice, for the number of PV+ cells, a 2-way ANOVA indicated a significant interaction between stress and timing of stress (F_1,28_ = 5.14, *P* = .03) and a trend for a stress effect (F_1,28_ = 3.80, *P* = .06). Tukey post-test indicated that adolescent stress decreased the number of PV+ cells (*P* < .05 vs naïve of their respective group; Figure 5A). Regarding PNN+ cells, a 2-way ANOVA revealed a trend for a stress effect (F_1,28_ = 3.63, *P* = .07) and an effect of timing of stress (F_1,28_ = 118.1, *P* < .0001) and interaction between these factors (F_1,28_ = 22.80, *P* < .0001). Post-hoc analysis revealed that PNN+ cell number decreased in adolescent-stressed male mice (*P* < .05 vs naïve; Tukey post-test; Figure 5B). Also, naïve animals from the experiment with adult stress (evaluated at PND115) presented a higher number of PNN+ cells than naïve animals from the experiment with adolescent stress (evaluated at PND80) (*P* < .05; Turkey post-test). While no changes were observed in PNN intensity when applying a 2-way ANOVA analysis, a significant decrease was found when the Student *t* test was performed comparing naïve vs adolescent stressed male mice (supplementary Figure 2A). For PV+/PNN+ colocalization, there were effects of stress (F_1,28_ = 10.08, *P* = .004), timing of stress (F_1,28_ = 43.02, *P* < .0001), and interaction between them (F_1,28_ = 4.61, *P* = .04). PV+/PNN+ colocalization decreased in the PFC of male mice exposed to adolescent stress (*P* < .05; Turkey post-test; Figure 5C). As observed for PNN+ cell number, naïve animals in the adult stress experiment showed a higher number of PV+/PNN+ colocalization compared with naïve mice in the adolescent stress experiment (*P* < .05; Turkey post-test). These findings suggest a complex interplay between stress, timing of stress, and neuroplasticity-related markers in male mice, highlighting the importance of considering these factors in understanding stress-related alterations in the brain.

In female mice, stress exposure during adolescence and adulthood did not impact PV+ (Figure 5D) and PNN+ cell numbers (Figure 5E) but tended to decrease PNN intensity (supplementary Figure 2B). In contrast, for PV+/PNN+ colocalization (Figure 5F), a 2-way ANOVA implied a significant effect for stress (F_1,32_ = 19.93, *P* < .0001), timing of stress (F_1,32_ = 27.86, *P* < .0001), and an interaction between them (F_1,32_ = 5.98, *P* = .02). Tukey post-test indicated that adolescence stress in female mice impacted PV+/PNN+ colocalization (*P* < .05 vs naïve of their respective groups). These results suggest a significant interplay between stress and developmental factors on PV and PNN markers in the PFC of female mice.

**Figure 6.**
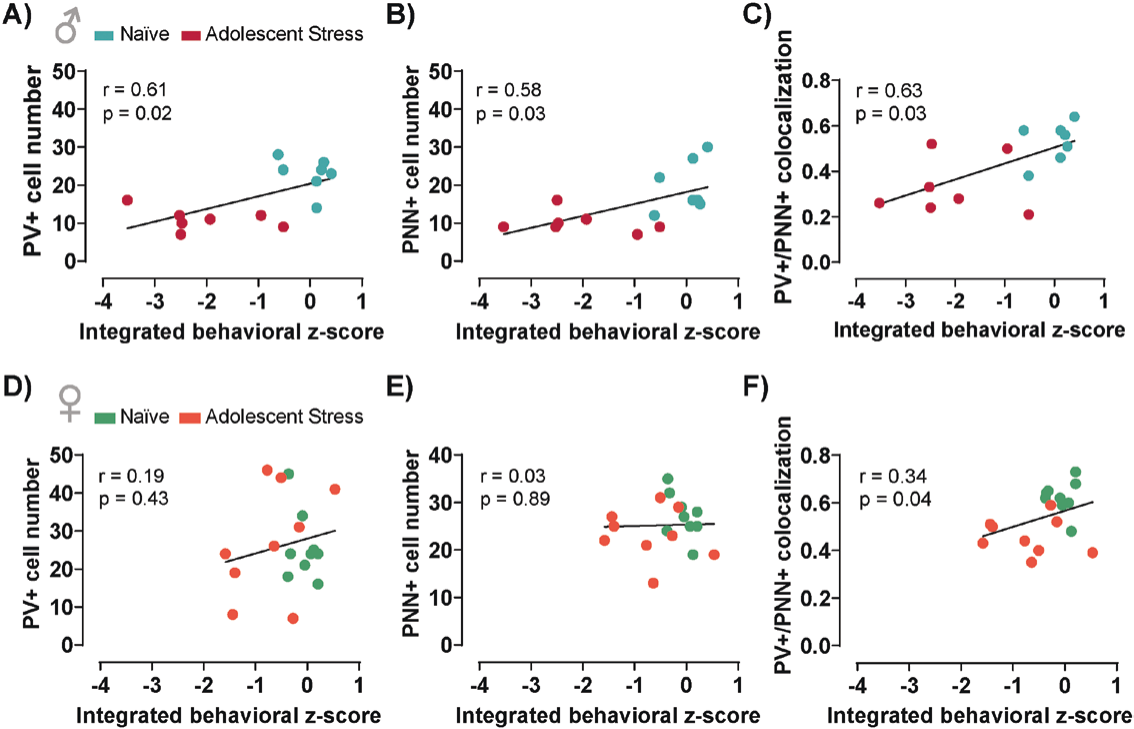
Association between the overall behavioral performance of male and female mice exposed or not to adolescent stress with changes in parvalbumin (PV)+ and perineuronal nets (PNN)+ cell numbers and PV+/PNN+ colocalization in the prefrontal cortex (PFC). For adolescent-stressed male mice and their naïve counterparts, positive correlations were found between the integrated behavioral z-score and (A) PV+ cell number, (B) PNN+ cell number, and (C) PV+/PNN+ colocalization. In adolescent-stressed female mice and its respective control group contrast, (D) PV+ and (E) PNN+ cell numbers were not correlated with the integrated behavioral z-score. However, (F) Pearson correlation analysis indicated a positive correlation between integrated behavioral z-score and PV+/PNN+ colocalization in the PFC of female adolescent stressed and naïve animals. r and *P* values are displayed in the graphs.

Then, we performed a Pearson correlation analysis to evaluate the integrated behavioral z-score performance of male and female mice (Figure 4A) with the association with PV+ and PNN+ cell numbers and PV+/PNN+ colocalization (Figure 5A–F). In male mice subjected to adolescent stress and their corresponding naïve counterparts, the integrated behavioral z-score exhibited a positive correlation with PV+ and PNN+ cells and PV+/PNN+ colocalization, suggesting that male mice exposed to adolescent stress displaying inferior behavioral performance tend to have lower levels of PV+ and PNN+ cells (Figure 6A–C). In female mice exposed to adolescent stress and their naïve counterparts, PV+ and PNN+ cell numbers lack correlation with the integrated behavioral z-score (Figure 6D–E). However, PV+/PNN+ colocalization positively correlated with the integrated behavioral z-score (Figure 6F), suggesting a specific impact of adolescent stress on female neural circuits underlying the overall behavioral performance. No significant correlations were found in adult-stressed male and female mice (supplementary Figure 3A–F).

### Sex-Specific Variability in Response to Adolescent Stress

**Figure 7.**
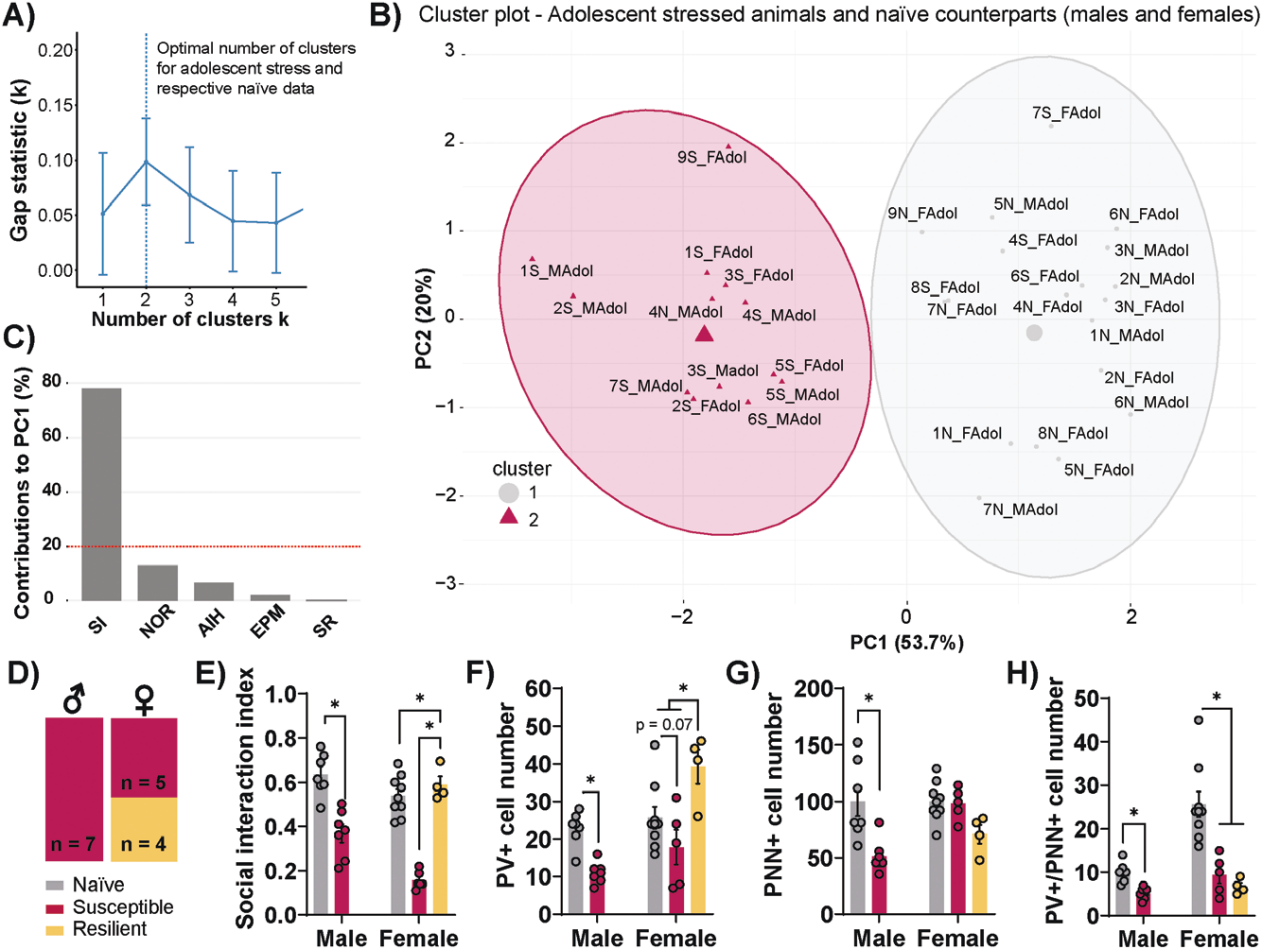
Female mice cluster into different groups based on the behavioral adolescent stress response. (A) The optimal number of clusters determined by GAP statistical methods is k = 2. (B) Expression of significant principal components (PCs) considering all behavioral paradigms. Dots represent integrated z-scores from the behavioral paradigms for each mouse. Two groups of individuals, gray (cluster 1) and red (cluster 2) dots, are separated by k-means clustering. (C) Social interaction (SI) index reached 78% of the contribution to PC1, while the other behavioral parameters did not achieve the minimum 20% of the contribution. (D) Cluster 1 was composed of male and female naïve animals and partial of the female mice exposed to adolescent stress (n = 5), in which were considered “resilient” to stress effects. Cluster 2 was composed of all (n = 7) male mice exposed to adolescent stress were “susceptible” to our stress protocol, and the remained female mice (n = 4) exposed to adolescent stress, in which were categorized as “susceptible” to stress. (E) Adolescent stress reduced the SI index in both male and female “susceptible” groups compared with their naïve counterparts. “Susceptible”-stressed female mice showed diminished SI index compared with “resilient”-stressed female mice. (F) The percent of parvalbumin (PV)+ cell number (relative to control) was reduced in “susceptible”-stressed male mice compared with the respective naïve group. Although the percent of PV+ cells between “susceptible”-stressed females and their naïve counterparts tends to decrease, the “resilient”-stressed female group displayed an increased percent of PV+ cell number compared with the “susceptible”-stressed female and naïve female groups. (G) In “susceptible”-stressed male mice, there is a decrease in the percent of perineuronal nets (PNN)+ cell number compared with their naïve counterparts, while “susceptible”- and “resilient”-stressed females remained like in naïve animals. (H) The percent of PV+/PNN+ colocalization was decreased in male mice “susceptible” to adolescent stress and in the “resilient”-stressed female animals compared with their respective naïve groups. Data are shown as mean ± SEM. *P < .05; 1-way ANOVA followed by Tukey post-test.

To delve further into the interindividual variability of stress response in male and female mice, we conducted a clustering analysis followed by principal component analysis (PCA) on the z-normalized behavioral scores. The unsupervised k-mean clustering analysis (GAP statistic method) indicated k = 2 as the optimal number of clusters for adolescent stress behavioral data (Figure 7A). On the other hand, the optimal number of clusters for adulthood behavioral data was k = 1 (supplementary Figure 4), implying that animals stressed during adulthood showed the same behavioral output as naïve animals. Therefore, we performed PCA including only male and female mice exposed to adolescent stress and their respective naïve groups. Based on the first and second principal components (PC), male and female mice exposed to adolescent stress, along with their corresponding naïve groups, were categorized into 2 distinct groups (Figure 7B). Cluster 1 was composed of naïve male and female mice, as well as by 4 adolescent stressed female mice, while cluster 2 comprised adolescent stressed male mice (n = 7) and 5 adolescent stressed female animals. The highest contributor to the PC1, which accounted for 53.7% of the explained variance, was the SI index (78%) (Figure 7C).

Given that certain adolescent-stressed female mice (n = 4) clustered alongside naïve animals of both sexes, we classify this subgroup of females as “resilient” to stress. Conversely, the remaining adolescent-stressed female animals (n = 5) clustered together with adolescent-stressed male mice (n = 7), leading us to categorize them as “susceptible” to stress (Figure 7D). Indeed, 1-way ANOVA indicated a stress effect in the sociability index (F_4,27_ = 26, *P* < .0001). As expected, “susceptible”-stressed male and female groups presented a decreased SI index compared with their respective naïve counterparts (*P* < .0001; Tukey post-test). Also, the SI index of “susceptible”-stressed female mice was reduced in comparison to the “resilient”-stressed female group (*P* < .0001; Tukey post-test; Figure 7E). Notably, “susceptible”-stressed female mice did not show changes in anxiety-like behaviors, social discrimination, cognitive function, and dopamine system responsivity (supplementary Figure 5A–F), further supporting the SI index as the main variable to cluster female subgroups.

To compare the magnitude of stress-induced effects on “susceptible” and “resilient” male and female animals concerning the PFC neuroplastic markers, we evaluated the percent of PV+ and PNN+ cell numbers and PV+/PNN+ colocalization relative to the naïve groups. One-way ANOVA indicated an effect of stress in the percent of PV+ cell number (F_4,27_ = 9.36, *P* < .0001), with a reduction in the percent of PV+ cells in “susceptible”-stressed male mice (*P* = .02; Tukey post-test) and a trend to decrease it in “susceptible”-stressed females (*P* = .07; Tukey post-test). In addition, the percent of PV+ cell number in the PFC of “resilient”-stressed female mice increases compared with the “susceptible”-stressed female (*P* = .002; Tukey post-test) and naïve groups (*P* = .04; Tukey post-test; Figure 7F). Regarding the percent of PNN+ cell number, a significant effect of stress was indicated by 1-way ANOVA (F_4,27_ = 7.19, *P* = .0004). In “susceptible”-stressed male mice, the percent of PNN+ cell number was reduced (*P *= .002; Tukey post-test), whereas in “susceptible”- and “resilient”-stressed female mice, it remained similar to naïve animals. Additionally, a stress effect was found for the percent of PV+/PNN+ colocalization (F_4,27_ = 9.5, *P* < .0001), which was decreased in male “susceptible” to adolescent stress (*P *= .001; Tukey post-test) as well as in the “resilient”-stressed female animals (*P *= .004; Tukey post-test; Figure 7H). These findings suggest that stress during adolescence has differential effects on female mice, impacting sociability and the magnitude of PV+ and PNN+ cell numbers and PV+/PNN+ colocalization in the PFC, with putative implications for understanding stress resilience and susceptibility in this population.

## DISCUSSION

Understanding how stress manifests differently throughout life stages and between sexes offers valuable insights into developing targeted therapeutic approaches to ameliorate stress-related psychiatric disorders. Here, we showed that adolescent stress in male mice elicited anxiety-like behaviors, loss of sociability, cognitive deficits, and altered dopamine system responsivity in adulthood. In addition, these behavioral deficits correlated with reductions in PV+ and PNN+ cells in the PFC, evidencing the importance of sensitive periods of neuroplasticity in the emergence of stress-induced long-lasting changes in different behavioral domains that are altered in several psychiatric disorders. Furthermore, we revealed sex-specific divergences in stress response, in which male mice appeared to be more severely affected by adolescent stress, as indicated by a larger decrease in the integrated z-scores compared with females exposed to adolescent stress. By employing PCA, we identified distinct behavioral clusters in which all male mice exposed to adolescent stress were clustered together and, therefore, were considered “susceptible.” In contrast, some female mice displayed “resilience,” and others were “susceptible” to adolescent stress-induced sociability deficits, along with decreased prefrontal PV+ cells. These findings underscore the importance of considering the timing of stress and sex differences in stress research and highlight the need for further investigation into individual variability in stress response.

Adolescence is a period of heightened susceptibility to stressors since the brain undergoes significant neuroplasticity by inducing structural and functional changes, which remain until early adulthood (Romeo, 2013). In contrast, the adult brain has already completed the developmental process, making it less susceptible to stress and more capable of adapting (Gee and Casey, 2015). A meta-analysis revealed a higher median age at mental disorder onset during the transitional period across adolescence and young, evidencing that some psychiatric disorders, such as anxiety- and fear-related disorders and schizophrenia, often originate during the early phases of neurodevelopment and typically reach their manifestation peak by mid- to late adolescence (Solmi et al., 2021). Here, stress exposure during adolescence, but not adulthood, elicited a spectrum of long-lasting behavioral changes in male mice, including anxiety-related behaviors, diminished sociability, cognitive deficits, and heightened responsivity to amphetamine. Consistent with these findings, our previous studies in rats demonstrated similar behavioral alterations at 1–2 and 5–6 weeks post-adolescent stress. However, no differences were observed in response to stress during adulthood (Gomes and Grace, 2017; Gomes et al., 2020). Although we did not specifically assess depression-related behaviors, in our previous studies, adult stress induced a hypodopaminergic state in the ventral tegmental area 1–2 weeks post-stress, as observed in animal models for depression (Moreines et al., 2017; Uliana et al., 2020), which normalized by 5–6 weeks post-stress (Gomes et al., 2020; Uliana et al., 2022).

Our study applied the integrated behavioral z-score approach to compare stress responses between male and female mice across different behavioral domains, including anxiety, sociability, cognition, and dopamine system responsivity. Specifically, adolescent stress led to impairments in integrated behavioral z-scores in both males and females, indicating a detrimental impact of stress on behavior. Importantly, male mice were more affected by adolescent stress than females, highlighting sex-specific differences in stress response. Female mice lacked stress-induced behavioral changes when raw data were analyzed, which contrasts with studies reporting increased susceptibility to stress-related disorders and depression in women compared with men (Bangasser and Valentino, 2014; Olff, 2017; Mengelkoch and Slavich, 2024). Several reasons can explain why significant changes in females’ responses to stress may not be observed (Mengelkoch and Slavich, 2024). For instance, female rats demonstrate susceptibility to combined footshock and restraint stressors when exposed later during adolescence (PND41–50) but not in prepuberty (PND21–30) and early adolescence (PND31–40) (Klinger et al., 2019; Zhu and Grace, 2023). Therefore, the timing of stress exposure used in the current study may not have been adequate to elicit measurable, long-lasting behavioral changes in female mice. Furthermore, females may exhibit a faster or more complete recovery from the stress effects than males. For instance, female rats exposed to early adolescent and adult stress displayed a short-term, that is, 1–2 weeks post-stress, hypodopaminergic state (Klinger et al., 2019) like that found in animal models for depression, such as the chronic mild stress and learned helplessness paradigms (Chang and Grace, 2014; Rincón-Cortés and Grace, 2017). Considering that we evaluated the behavioral domains of female mice 5 weeks after adolescent and adult stress, it is speculative that the initial effects of stress might have recovered by the time of testing.

Stress-induced changes require careful consideration of methodological factors, including the timing, duration, and intensity of stress exposure. However, it is equally crucial to recognize the inherent variability in stress response among individuals (Jakovcevski et al., 2008; Castro et al., 2012; Larrieu et al., 2017; Zalachoras et al., 2022). Using PCA, we found that some female mice clustered alongside naïve counterparts and others grouped with adolescent stressed counterparts, suggesting differential susceptibility to stress-induced behavioral changes. The sociability index emerged as a key contributor to the separation of clusters, indicating its importance in distinguishing between “susceptible” and “resilient” individuals. While the sample size of our study is sufficient to provide preliminary insights into stress resilience, future studies can build on these findings by increasing the number of animals to validate and extend the results. Nonetheless, our findings align with previous research elucidating individual variability in stress response and resilience to adverse experiences in animal models and human studies (Cathomas et al., 2019). For example, individual differences in response to adult chronic unpredictable stress revealed that some mice exhibit resilience to the emergence of depression- and anxiety-related behaviors (Nasca et al., 2015; Brivio et al., 2022). These insights into stress response variability and resilience are crucial to uncovering neurobiological mechanisms of individual susceptibility.

The prelimbic subregion of PFC is pivotal in regulating cognitive functions and emotional responses, including stress responsivity, by inhibiting stress-evoked responses in the amygdala and hippocampus (Granon and Poucet, 2000; Rosenkranz and Grace, 2002; Rosenkranz et al., 2003; Quirk and Beer, 2006). Disruptions in the prelimbic cortex are linked to changes in behavior, such as anxiety, social recognition, and cognitive impairments (Gomes and Grace, 2017; Uliana et al., 2020; Yashima et al., 2023). PVIs in the prelimbic PFC are central to establishing the timing and duration of periods of plasticity, including adolescence, marked by enhanced neuroplasticity and heightened vulnerability to stress. As neurodevelopment progresses, molecular regulators like PNNs wrap PVIs to close critical periods of plasticity (Reichelt et al., 2019). PNNs act as a protective barrier for PVIs, shielding them from metabolic and oxidative harm (Cabungcal et al., 2013; Steullet et al., 2017). PNN formation typically concludes in early adulthood, making PVIs more susceptible to stressful situations before this period (Ruden et al., 2021). Indeed, PNN degradation in adult animals recreates an adolescent-like phenotype of stress susceptibility (Colodete et al., 2024). Here, in male mice, we found that naïve animals from the experiment with adult stress (evaluated at PD 115) presented more PNN+ cells and PV+/PNN+ colocalization than naïve animals from the experiment with adolescent stress (evaluated at PD 80). This was unexpected, given that, despite age differences, both groups were assessed when PNNs were supposed to be mature and completely formed. In contrast to the literature (Page and Coutellier, 2018), we also found sex differences in PV+ and PNN+ cells and PV+/PNN+ colocalization, indicating that adult female mice presented higher levels of cortical PV+ and PNN+ cells.

Regarding the impact of stress, adolescent stress reduced PV+ and PNN+ cells and PV+/PNN+ colocalization in the PFC of male mice, indicating disrupted interaction post-stress. Conversely, male mice exposed to adult stress showed no significant changes in PV+ and PNN+ cells, supporting that the immaturity of PVIs during adolescence may underlie their vulnerability to stress. In females, adolescent stress caused no significant change in the number of PV+ and PNN+ cells but decreased PV+/PNN+ colocalization, suggesting, once again, distinct stress effects between males and females. Regarding PV+ cells, our findings are similar to those found in adult male and female mice subject to a chronic unpredictable stress protocol during adolescence (PND28–42), with males showing fewer PV+ cells in the PFC and females with no change (Page and Coutellier, 2018). Evidence indicates that sex-specific factors, including hormonal fluctuations and genetic differences, may contribute to stress susceptibility and resilience variations between males and females (Ellis and Honeycutt, 2021). For instance, female mice experience a progressive rise in PV expression levels during adolescence, influenced by sex steroid hormone levels, a phenomenon not observed in males (Ross and Porter, 2002; Wu et al., 2014). Therefore, it is possible that sex hormone influences on PVIs during earlier developmental stages, unlike in males, may contribute to the absence of behavioral changes observed in females following adolescent stress.

Cortical PVIs and PNNs are implicated in stress resilience (Perova et al., 2015; Bhatti et al., 2022; Favoretto et al., 2023). In our study, female mice exposed to adolescent stress who exhibited “resilience” to sociability impairments showed normal PV labeling, whereas those displaying “susceptibility” exhibited reduced PV+ cells. This finding is consistent with evidence indicating that resilience to behavioral effects of chronic stress is mediated by differential neuronal activity and gene expression in PVIs (Bhatti et al., 2022). Furthermore, in the learned helplessness paradigm, activation of prefrontal PVIs during stress promotes the establishment of resilient behavior (Perova et al., 2015). Therefore, targeting PVI activity could offer novel strategies for enhancing stress resilience and mitigating the adverse effects of stress-related psychiatric disorders. For example, we previously showed that pharmacological agents that could enhance PVI inhibitory neurotransmission attenuate adolescent stress-induced behavioral and electrophysiological changes (Cavichioli et al., 2023).

Although the number of PNN+ cells in “susceptible”- and “resilient”-stressed female mice remained similar to the naïve group, the amount of PVIs wrapped by PNNs decreased in both female subgroups. PNNs have been predominantly studied in association with PVIs. However, circumvent other neuron subtypes, such as excitatory pyramidal neurons (Lensjø et al., 2017). Therefore, it is speculative that the lack of changes in PNN+ cell number in “susceptible”- and “resilient”-stressed female groups may rise from PNNs around different types of PFC neurons, potentially impacting synaptic function and plasticity in diverse ways (Carstens et al., 2016; Morikawa et al., 2017). Furthermore, stress can disrupt PNN molecular composition, such as the expression of aggrecan and neurocan, and their associated plasticity-related molecules without impacting the PNN+ cell number (Koskinen et al., 2020; Bueno-Fernandez et al., 2021; Laham and Gould, 2021). Additional studies are needed to unravel the complex interplay between stress, neuroplasticity, and sex-specific differences and to identify therapeutic targets for enhancing stress resilience.

Although this study highlights the age-dependent effects of stress, it is crucial to consider that the intensity of the footshock protocol might differentially impact adolescent and adult mice due to their varying body sizes and developmental stages (Yezierski, 2012). For instance, younger animals are more sensitive to electric shocks (Hess et al., 1981) and have a lower pain threshold overall (Lautenbacher et al., 2017), which might aggravate the adolescent stress reaction. To address this, future studies are needed to determine the minimum shock intensity that induces a stress response in adolescent and adult mice and potentially to adjust the stress protocol accordingly for age-related differences.

Overall, our study underscores the critical impact of stress on neurobehavioral outcomes, particularly during adolescence, a period marked by heightened vulnerability to stress-induced alterations. Our findings highlight the importance of considering age and sex differences in stress research and emphasize the need for further investigation into individual variability in stress response. Advancing our knowledge of stress response holds promise for the development of personalized interventions aimed at mitigating the adverse effects of stress-related psychiatric disorders and promoting resilience across diverse populations.

## Supplementary Material

pyae042_suppl_Supplementary_Figures_S1-S5

## Data Availability

The data underlying this article will be shared upon request.
