# Supplementary material for: Differential Impact of Adolescent or Adult Stress on Behavior and Cortical Parvalbumin Interneurons and Perineuronal Nets in Male and Female Mice
Source: Int J Neuropsychopharmacol. 2024 Sep 14;27(10):pyae042. doi: 10.1093/ijnp/pyae042 (PMC11639180; doi:10.1093/ijnp/pyae042)
Supplement: pyae042_suppl_Supplementary_Figures_S1-S5 [file pyae042_suppl_supplementary_figures_s1-s5.docx]

**Differential impact of adolescent or adult stress on behavior and cortical parvalbumin interneurons and perineuronal nets in male and female mice**

Thamyris Santos-Silva^1^, Beatriz Kinchin Souza^1^, Débora Akemi Endo Colodete^1^, Lara Ramos Campos^1^, Thaís Santos Almeida Lima^1^, Francisco S. Guimarães^1^, Felipe V. Gomes^1^*

1Department of Pharmacology, Ribeirão Preto Medical School, University of São Paulo, Ribeirão Preto, Brazil

*Corresponding author: Felipe V. Gomes, Department of Pharmacology, Ribeirão Preto Medical School, University of São Paulo, Avenida Bandeirantes 3900, Ribeirão Preto, SP, 14049-900, Brazil. Email: gomesfv@usp.br.

***Supplemental Information***

[EXTEND METHODS 2](#_Toc174732531)

[Animals 2](#_Toc174732532)

[Stress Protocol 2](#_Toc174732533)

[Behavioral tests 2](#_Toc174732534)

[Elevated plus maze (EPM) 2](#_Toc174732535)

[Social interaction (SI) and social discrimination (SD) 3](#_Toc174732536)

[Novel object recognition (NOR) 3](#_Toc174732537)

[Amphetamine-induced hyperlocomotion (AIH) 4](#_Toc174732538)

[Immunofluorescence 4](#_Toc174732539)

[Statistical analyses 5](#_Toc174732540)

[SUPPLEMENTARY FIGURES 6](#_Toc174732541)

[Supplementary Figure 1 6](#_Toc174732542)

[Supplementary Figure 2 7](#_Toc174732543)

[Supplementary Figure 3 8](#_Toc174732544)

[Supplementary Figure 4 9](#_Toc174732545)

[Supplementary Figure 5 10](#_Toc174732546)

# EXTEND METHODS

# Animals

Male and female C57BL/6 mice (postnatal day, PND 24 or 58) were obtained from the colony maintained by the Central Animal House of the University of São Paulo (USP), campus Ribeirão Preto. Animals were randomly assigned to experimental groups, each cage devoted to a specific experimental procedure. Mice were randomly housed (2 – 4 animals per cage) at temperature- (22°C) and humidity-controlled (47%) room. Experiments started one week after they arrived in the Central Animal House of the Department of Pharmacology, Ribeirão Preto Medical School, USP. The Ribeirão Preto Medical School Ethics Committee (# 100/2019) approved the procedures following Brazilian and international regulations.

# Stress Protocol

Adolescent mice were exposed to daily inescapable footshock (FS) from PND 31 to 40. Similarly, changes induced by FS exposure were also evaluated in adulthood (from PND 65 to 74). Briefly, mice were exposed to one session of FS per day for ten consecutive days. In each session, animals were placed in a Plexiglas chamber with a grid floor of 0.23 cm stainless steel rods spaced 1.0 cm apart (Insight Equipment, Brazil). Fifteen FS (0.75 mA, 2 s) was delivered pseudo-randomly (5 cycles of 30, 60, 40, 60, and 90 seconds) in each session. Naïve animals were left undisturbed in their home cages, while stressed animals were subjected to the stress protocol.

# Behavioral tests

## ***Elevated plus maze (EPM)***

The EPM consisted of two opposite wooden open arms (30 × 5 cm) crossed at a right angle by two closed arms (30 × 5 × 15 cm). The maze was located 50 cm above the floor, and a 1 cm high edge made of Plexiglas surrounded the open arms to prevent falls. Animals were placed on the central platform with the head facing one of the enclosed arms to explore the EPM freely for 5 minutes (min). The percentage (%) of open arms entries, time spent in these arms, and the number of entries in the enclosed arms were recorded using the Any-maze software (Stoelting, USA).

## ***Social interaction (SI) and social discrimination (SD)***

Animals were previously habituated for 5 min in an acrylic box containing three similar compartments and two empty cages. During the SI phase, a non-familial mouse was placed in a cage (social), while the other cage remained empty (non-social). The test animal explored the environment freely for 10 min. After the SI phase, the test mouse was confined in the middle compartment for 1 min before the SD phase started. The animal previously considered unfamiliar was now regarded as familiar, and a new mouse was placed in the other (non-familiar) cage. Then, the test animal explored the environment for 5 min during the SD phase. The results were reported as SI index (SII) or SD index (SDI):

SII = [(t_social_ – t_non-social_)/( t_social_ + t_non-social_)]

SDI = [(t_non-familiar_ – t_familiar_)/( t_non-familiar_ + t_familiar_)]

## ***Novel object recognition (NOR)***

Two hours after the SD phase, each animal was subjected to an open field (OF), consisting of a circular arena (30 × 30 cm), for 10 min (OF test). The total distance traveled and the distance traveled in the center zone were recorded using the Any-maze software. The NOR test was conducted in the same circular arena 24 hours (h) later. Animals were subjected to two trials separated by 1 h. During the first trial (acquisition trial, T1), mice were placed in the arena containing two identical objects for 10 min. For the second trial (retention trial, T2), one of the objects presented in T1 was replaced by an unknown (novel) object. Animals were then placed back in the arena for 10 min. Object exploration was defined as when the animal faced the object at 2 cm of distance or less while watching, licking, sniffing, or touching it with the forepaws while sniffing. A blind experimenter quantified object exploration. Recognition memory was assessed using the following index: [(t_novel object_ – t_familiar object_)/( t_novel object_ + t_familiar object_)].

## ***Amphetamine-induced hyperlocomotion (AIH)***

Basal locomotor activity was recorded for 30 min. After that, mice were injected with saline (1 mL/Kg) or D-amphetamine sulfate (1 mg/Kg, i.p.; Sigma), and their locomotor activity was recorded for another 40 min.

# Immunofluorescence

Two days after the AIH, mice were deeply anesthetized with urethane 25% (5 mL/kg, intraperitoneal) and transcardially perfused with 0.01 M phosphate-buffered saline (PBS, pH=7.6), followed by 4% PFA in 0.01 M PBS (pH = 7.6). Then, brains were removed, post-fixed in 4% PFA for 2 h, and stored in 30% sucrose. Serial 30 μm-thick coronal sections of the PFC were collected using a cryostat (CM-1900, Leica). For each animal, five to six sections spanning the rostrocaudal axis of the PFC (containing the prelimbic region) were collected and stained. Specifically, sections were incubated in a combination of 1% normal goat serum, 0.1% Triton X-100, rabbit anti-PV antibody (1:1000, Swant, PV 25), and biotinylated Wisteria floribunda agglutinin (WFA; 1:500 dilution, Vector Labs, #B1355) for 24 h at 4°C. The sections were then incubated with a mixture of 1% normal goat serum, goat anti-rabbit Alexa Fluor 488 (1:500, Abcam, ab150077), and Alexa Fluor 594 conjugated to streptavidin (1:500, Abcam, ab272189) for 90 min. The sections were mounted with Fluoroshield Mounting Medium with DAPI (Abcam, ab104139) to visualize the border of the PFC. For image acquisition, the focus was set on PV-positive cells for imaging, and digital images were obtained using Leica Application Suite X (Leica Microsystems). Under 20x magnification, the PFC regions of six rostrocaudal sections were imaged by z-stacks (512 × 512 μm-images along the medial-temporal axis) using a confocal microscope (SP5, Leica). For cell count, only the prelimbic region was counted. The exposure time for PV was calibrated such that most of the PV+ cells in the naïve group were visible and within the dynamic range, and all subsequent images of the remaining age groups were taken at an identical exposure. PNNs were identified by staining for WFA, a lectin that selectively labels residues of glycoproteins within the PNNs. Counterstaining for PV enables us to tell if the structure is indeed a perisomatic PNN encompassing PV interneurons. For analysis, acquired images were first converted to maximum projection (z-stacks). The PV+ and PNN+ cell counts were performed using Fiji software, a cell counts plugin. The Manders overlap coefficient was used to analyze the colocalization of PV and PNNs, which measures the fraction of signal from channel 1 (PV+ label) that overlaps with channel 2 (PNN+ label).

# Statistical analyses

Data were presented as mean ± S.E.M and analyzed using one-, two-, or three-way ANOVA accordingly. Significant differences were indicated by p < 0.05. A Pearson correlation analysis tested the correlation between integrated behavioral z-score and the number of PV+, PNN+, and PV+/PNN+ colocalization. A clustering analysis followed by principal component analysis (PCA) on the z-normalized behavioral scores was performed to investigate interindividual variability of stress response in male and female mice. Statistical analyses were performed with Prism 9.0 (Graphpad Software Inc.) and R (R Core Team, 2014).

# SUPPLEMENTARY FIGURES

**
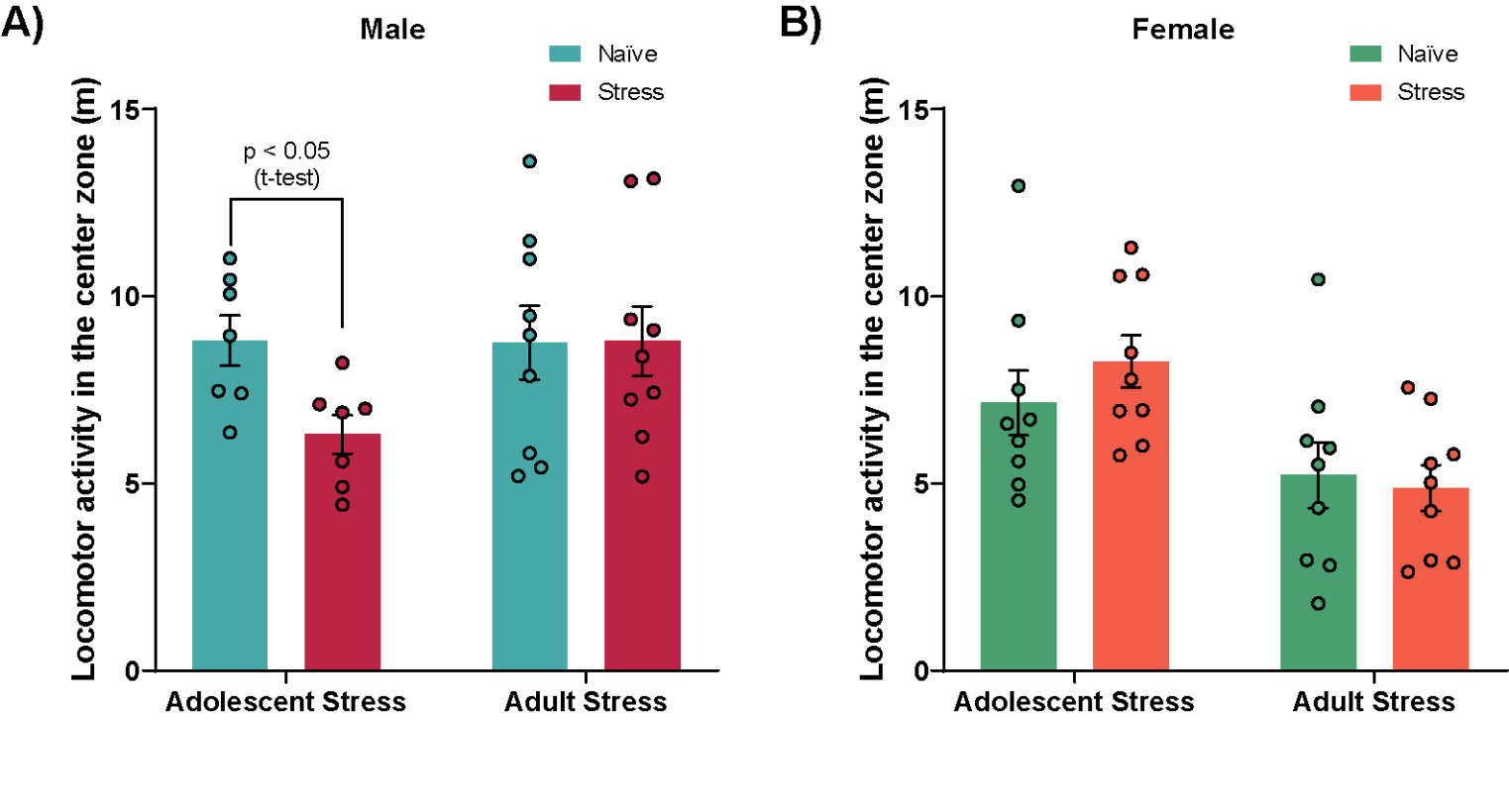
**

**Supplementary Figure 1. Effects of adolescent and adult stress on the locomotor distance in the center zone of the OF test.** Student-t test demonstrated that **(A)** adolescent-stressed male mice reduced the locomotor distance in the center zone (t_12_=2.96, p=0.01), whereas **(B)** no significant changes was observed in female mice stressed during adolescence or adulthood. In both sexes, Two-way ANOVA did not reveal significant changes in this parameter between groups. n=7-9/group. Data are presented as mean ± SEM; Student’s t-test.


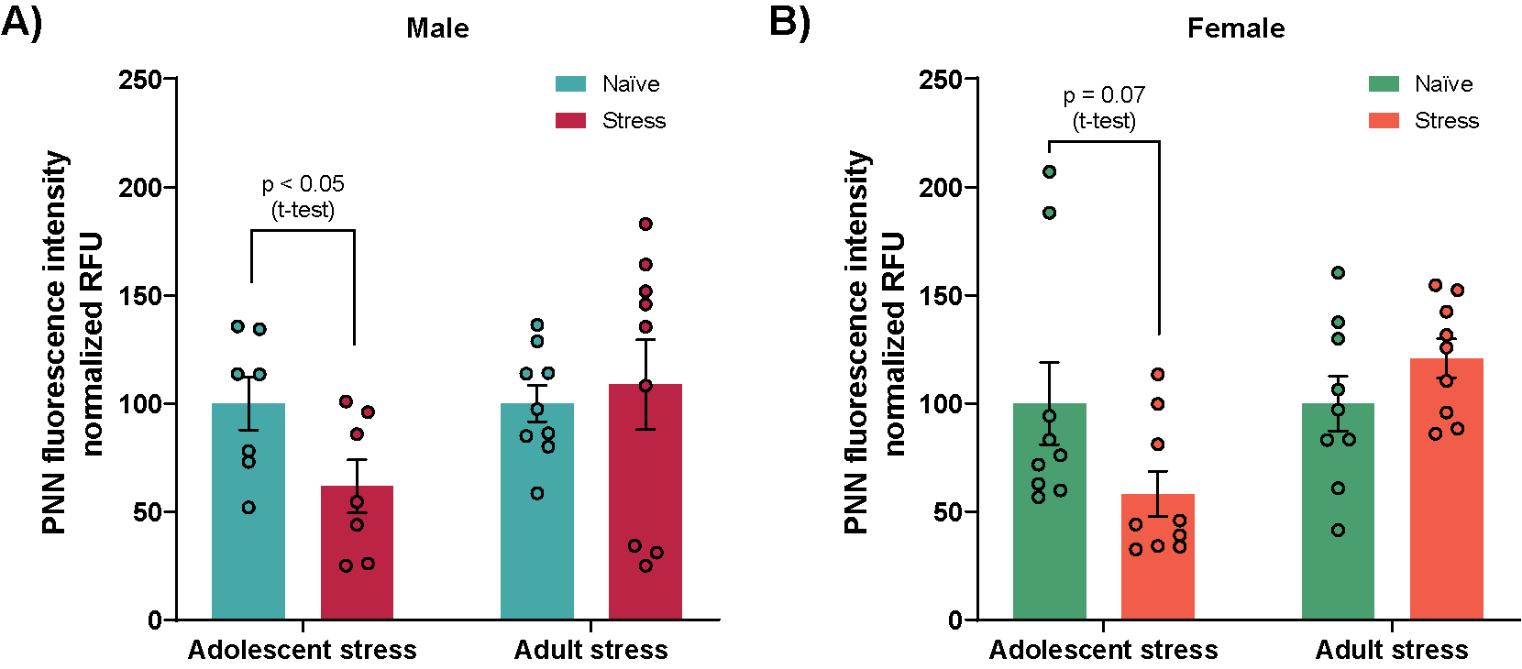


**Supplementary Figure 2. Effects of adolescent and adult stress on PNN fluorescent intensity.** By applying the Student’s t-test, **(A)** a significant decrease in the PNN intensity was observed when comparing naïve vs. adolescent stressed male mice (t_12_=2.22, p=0.04). **(B)** In female mice, there was a trend towards decreased PNN intensity after adolescent stress (t_16_=1.93, p=0.07). In both sexes, Two-way ANOVA did not reveal significant changes in this parameter between groups. n=7-9/group. Data are presented as mean ± SEM; Student’s t-test. RFU = relative fluorescent units.


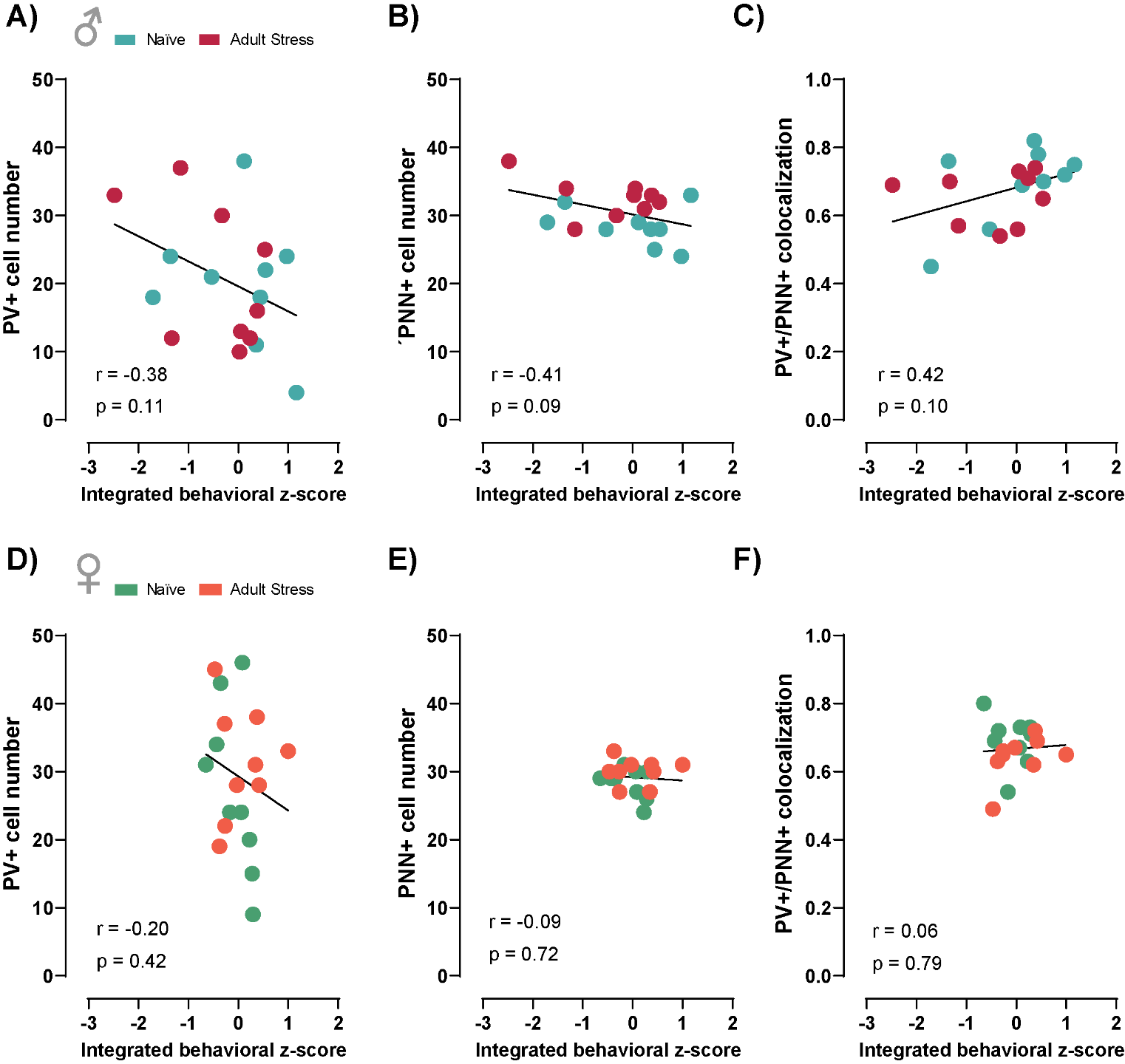


**Supplementary Figure 3.** No significant correlations between integrated behavioral z-score and PV+ cell number, PNN+ cell number and PV+/PNN+ colocalization were found in adult-stressed male **(A - C)** and female **(D - E)** mice and their respective control groups.

**
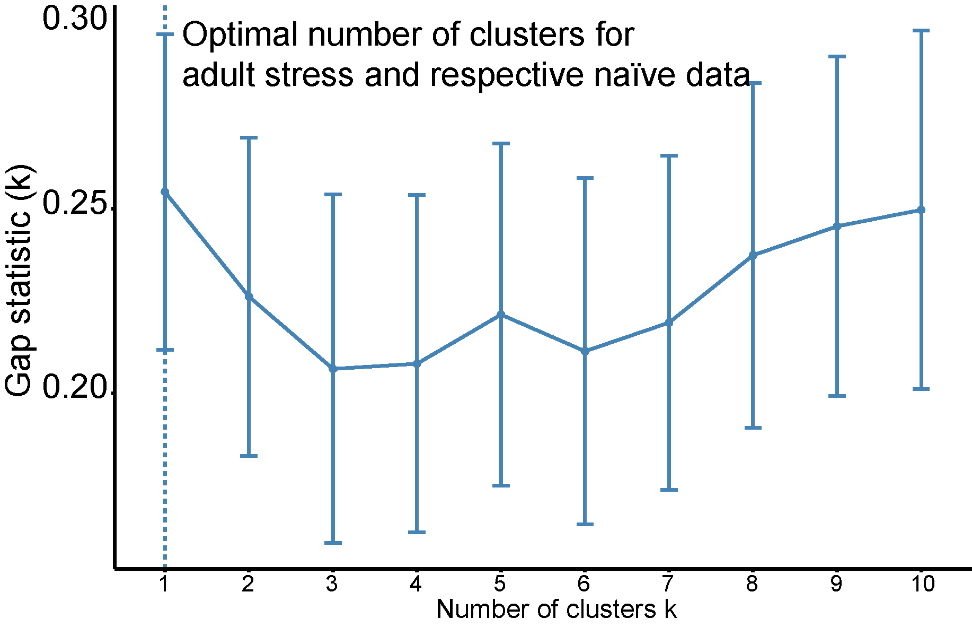
**

**Supplementary Figure 4.** The optimal number of clusters for adult stressed female and male mice and their respective naïve data was determined as k = 1.


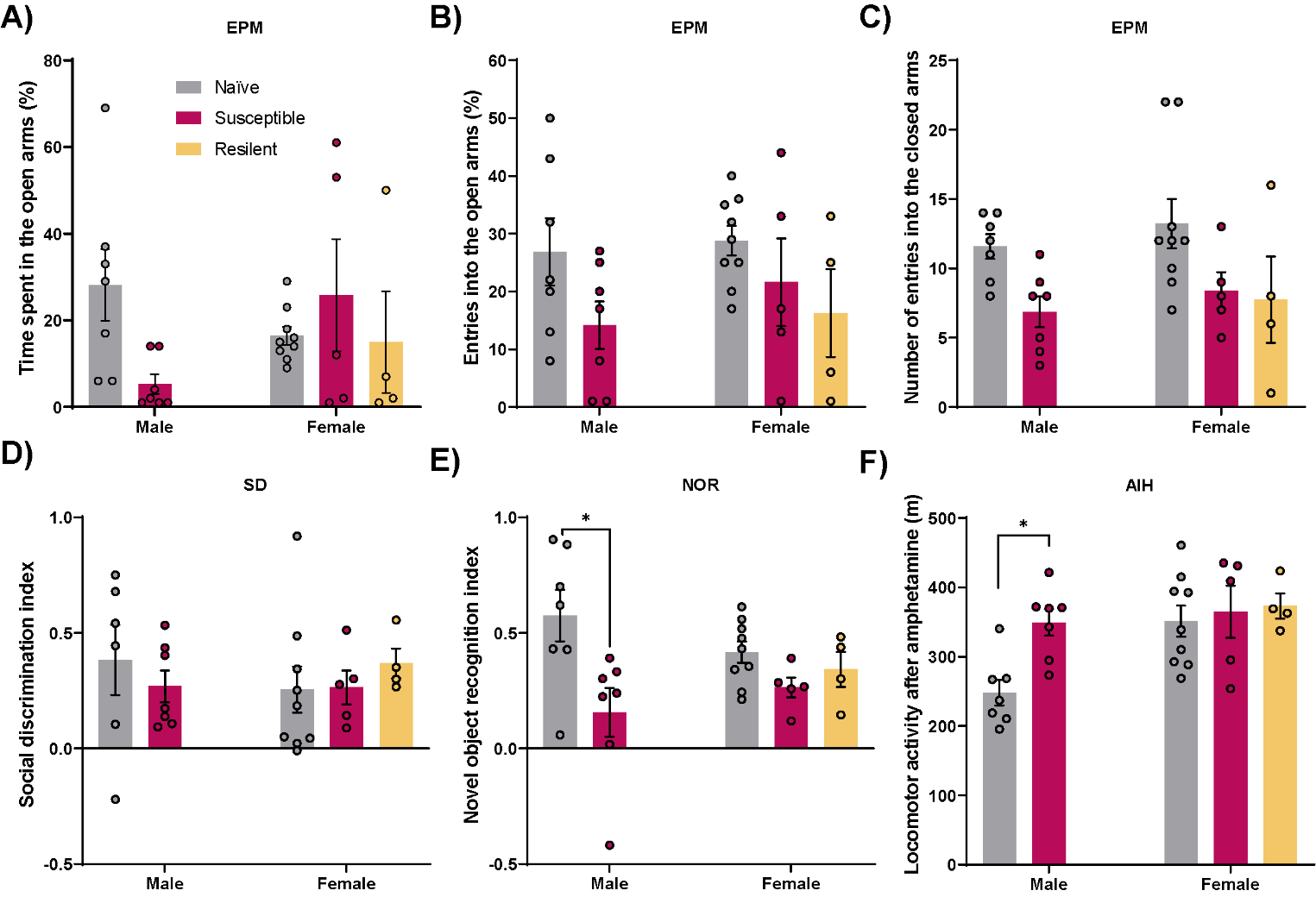


**Supplementary Figure 5.** No changes were found in **(A)** the % of time exploring the open arms, **(B)** the % of entries in the open arms and **(C)** in the number of entries in the closed arms of the elevated plus maze (EPM). **(D)** The social discrimination (SD) index also remained similar for all groups. **(E)** "Susceptible"-stressed male mice, but not "susceptible"- and "resilient"- stressed female mice, displayed reduced novel object recognition index (NOR) compared to their respective naïve group (F_4,27_=3.72, p=0.02). **(F)** Amphetamine-induced hyperlocomotion (AIH) was increased in "susceptible"-stressed male mice (F_4,27_=4.60, p=0.006), but not in "susceptible"- and "resilient"- stressed female mice. Data are shown as mean ± SEM or floating bars (line at mean; min to max). *p<0.05, one-way ANOVA followed by Tukey's post-test.
